# Methodologies to Evaluate the Hair Follicle-Targeted Drug Delivery Provided by Nanoparticles

**DOI:** 10.3390/pharmaceutics15072002

**Published:** 2023-07-21

**Authors:** Maíra N. Pereira, Luma L. Nogueira, Marcilio Cunha-Filho, Tais Gratieri, Guilherme M. Gelfuso

**Affiliations:** Laboratory of Food, Drug, and Cosmetics (LTMAC), School of Health Sciences, University of Brasilia, Brasilia 70910-900, DF, Brazil; mainunesp@gmail.com (M.N.P.); lumanogueira@unb.br (L.L.N.); marciliofarm@hotmail.com (M.C.-F.); tgratieri@gmail.com (T.G.)

**Keywords:** drug delivery, hair follicle, nanotechnology, skin model, topical delivery

## Abstract

Nanotechnology has been investigated for treatments of hair follicle disorders mainly because of the natural accumulation of solid nanoparticles in the follicular openings following a topical application, which provides a drug “targeting effect”. Despite the promising results regarding the therapeutic efficacy of topically applied nanoparticles, the literature has often presented controversial results regarding the targeting of hair follicle potential of nanoformulations. A closer look at the published works shows that study parameters such as the type of skin model, skin sections analyzed, employed controls, or even the extraction methodologies differ to a great extent among the studies, producing either unreliable results or precluding comparisons altogether. Hence, the present study proposes to review different skin models and methods for quantitative and qualitative analysis of follicular penetration of nano-entrapped drugs and their influence on the obtained results, as a way of providing more coherent study protocols for the intended application.

## 1. Introduction

Cutaneous topical administration of drugs and cosmetic actives presents numerous advantages for the treatment of dermatological diseases or conditions, such as fewer adverse effects related to systemic drug exposure, avoidance of the first-pass metabolism that impacts bioavailability and dose reduction, and targeted drug delivery [1,2]. However, topical therapies are always limited by the skin barrier function, mainly provided by the stratum corneum—the most superficial epidermal layer [3,4]. Following a topical application, the drug can diffuse through the skin cells or the extracellular matrix that composes the stratum corneum. Also, it can use the hair follicles as shunt pathways.

The hair follicle wall is an invagination of the epidermis containing a relatively constant composition of cells in the upper half of the hair follicle, infundibulum, and isthmus [5,6]. The hair follicle undergoes cyclical involution and regeneration throughout life. It is characterized as an epithelial organ consisting of two main parts: an epithelial cylinder composed of keratinocytes and the mesenchymal cells of the dermal papilla and dermal sheath. Structurally, the hair follicle is divided into four regions from top to bottom: infundibulum, isthmus, supra bulbar region, and medulla [7,8,9]. The composition of the lower region of the hair follicle is much more variable, including differentiated epithelial cells, hair matrix, and dermal papilla [10,11]. The hair bulb is situated at the base of each hair follicle and contains the growing hair cells. These cells are in a constant division that pushes upwards and gradually harden, reaching the highest part of the bulb and is organized by six concentric layers (three inner layers make up the hair, composed of the cuticle, the cortex, and the medulla and three outer layers make up the lining of the follicle) [12,13,14].

The follicular route as a shunt pathway was considered irrelevant in the past because of the limited surface area the hair follicles represent (0.1%) [15,16], with the maximum area of coverage found on the forehead (1.28%) and minimum on the forearm (0.09%) [17], apart from the scalp, where it represents 10% of the total area [18]. Nevertheless, this conception of being an irrelevant penetration route has completely changed with the advent of nanotechnology and the observation that solid nanostructures naturally accumulate into the hair follicles, opening valuable opportunities for topical treatments of diseases that precisely affect the follicles [6], such as hair growth disorders (areata and androgenetic alopecia) and other inflammatory processes that affect the follicular region, like acne or hidradenitis suppurativa [19,20]. In addition, other cutaneous diseases could be treated more effectively by targeting the hair follicles, such as hirsutism and hypertrichosis, as well as disorders like seborrhea, eczema, and rosacea [21].

Nanoparticulate systems have proved efficient in overcoming the skin barrier by diverse mechanisms, depending on their composition. Solid lipid nanoparticles, for instance, allow an efficient skin occlusion [22], with a consequent increase in the permeability of lipophilic drugs [23] or an increase in drug solubility, creating a higher concentration gradient that drives drug permeation through the stratum corneum [24]. Moreover, the low or complete absence of toxicity of this kind of nanoparticles [25], and the possibility of modified drug release make them exciting systems for topical drug delivery [26]. Other nanostructures, such as liposomes, can exert their function by adsorption and fusion of vesicles on the skin surface, stimulating the encapsulated drug to diffuse through the skin layers [27]. Liposomes also promote a drug delivery of much higher concentrations in the sebaceous glands than conventional formulations [28]. Parallel to this, the nanodroplets of nanoemulsions surrounded by surfactants operate as cutaneous permeation enhancers, favoring the skin penetration of topically applied drugs. However, generally, they do not demonstrate an impact on the follicular penetration of drugs because they are liquid systems [29,30].

The follicular drug penetration mechanism becomes a structurally complicated process concerning the factors that interfere with the follicular delivery of drugs [31,32]. The hair follicle delivery is influenced by the physicochemical characteristics of a drug (size and/or molecular weight of the drug and the oil–water partition coefficient), the size of the nanoformulation in which that drug is incorporated, which corroborates with a penetration into the depth and breadth of follicular delivery systems [33,34]. The nanoformulation can still present different compositions in its development, have surface loads that interfere with the interaction with the hair follicles and the skin, or even have a superficial modification in its structure according to the objective of the study [35,36].

Some studies have reported that the size of the solid nanoparticles influences the natural tendency of accumulation into the hair follicles, independent of the production materials. Nevertheless, once into the follicle, the type of material and its interaction with the drug has been demonstrated to play a role in the distribution process. Some investigations indicate that micrometer-sized particles [37,38] can permeate the hair follicles more efficiently than the nanoparticles themselves. Yet other studies demonstrate the feasibility of follicular absorption of nanosystems in the size range of 300–600 nm [39] or nanosystems with sizes less than 100 nm [40]. Analyzing this situation, the results seem controversial regarding the influence of particle characteristics in terms of composition and size range on the follicle accumulation process, making it difficult to draw conclusions on the best parameters for a targeted follicular delivery. Several studies cite and point to a survey of critical factors for greater targeting of follicles [41,42,43]. Nonetheless, experimental results often cannot be compared due to different methodologies employed in evaluating the nanostructured drugs’ follicular targeting.

In this way, this article proposes to review the main skin models that have been used to evaluate the targeted drug delivery to the hair follicles and the main in vitro methodologies (either qualitative or quantitative) used to assess the follicular targeting of drugs by nanostructured delivery systems aiming to obtain a better insight into the data produced so far.

## 2. Skin Models

In vitro and ex vivo skin permeation assays are crucial for developing new formulations for topical use. The first parameter that affects this type of study is the skin model to be selected, which presents various variabilities and influences on the entire methodological process. When follicular drug permeation is assessed, this parameter becomes crucial [44].

Human skin is an important model for evaluating topical formulations, obtained mainly from plastic surgeries or cadavers [45]. However, using human skin in permeability experiments comes up against limitations.

First, the ethical concern of employing human skin imposes severe restrictions for the experimental triggering. Additionally, much variability is encountered between specimens from different body locations, even from the same donor [45,46]. Another issue is the differences between individuals, possibly due to race, sex, skin thickness, and hydration. Nevertheless, a crucial point is that after the excision of human skin, the hair follicles suffer a contraction [47]. Therefore, this great structural change hinders the permeation of nanoparticle formulations targeting the follicular route, making the in vitro or ex vivo use of a human skin model inappropriate in such cases.

Parallel to the use of human skin, the skin of other animals has been used for studies, such as rabbits, snakes, rats, mice, and porcine [48]. Such skin types are more accessible models and have less variability due to using inbred animal lines [49]. Rabbit back skin has been tested in vitro for passive permeability. However, rabbit skin is more permeable [50], possibly due to the high density of hair follicles, of about 8000/cm^2^ [51].

Due to their availability, small size, and reasonable costs, rodents (e.g., rats and guinea pigs) are the most used animals for skin supply to in vivo skin permeation studies. Rat skin, indeed, holds structural similarities with human skin [49,52]. However, rat skin (stratum corneum thickness of 4.04 µm and viable epidermis thickness of 15.34 µm) is more permeable than human skin (stratum corneum thickness of 17 µm and viable epidermis thickness of 47 µm) through different permeants [53,54] due both to its smaller thickness compared to human skin and to its greater follicular density (1598 per cm^2^ for rat skin and 29 per cm^2^ for human skin) [55]. In addition, the number of appendages is greater, the intercellular lipid composition of the stratum corneum is different, and the surface of corneocytes is smaller than that of human skin. Accordingly, rat skin has been shown to be about 11 times more permeable than human skin, providing about 50 times greater flux for the relatively lipophilic molecules hydrocortisone and terbinafine [56]. Despite these differences, given the easy access to this type of skin model, rat skin can be helpful in comparing formulations. In addition, the model can be useful for formulations intended for application to the scalp for the comparable number of follicular units per square centimeter, which range between 65 and 85, with a hair density between 124 and 200 [57] and especially for specific protocols. For example, minoxidil penetration into the hair follicles was quantified in vitro after administering a commercial formulation (5% minoxidil solution) in rat and porcine skin. The study’s main objective was to evaluate the impact of wet or dry skin on drug absorption. With such a design, the use of the rat model made much more sense, as the presence of hair itself was a relevant parameter of the study. Such a study proved the humidity condition of the hair played a role in drug accumulation, with about five times greater retention in wet hair conditions [58].

However, porcine skin is perhaps the most used model for in vitro skin permeation studies [59,60,61,62,63,64], whether it is in studies considering the follicular route or not. It can be easily obtained from animals slaughtered for human consumption. An important measure, however, is to remove the desired skin part before the scalding process to guarantee its integrity. Also, as the skin would be used in the food process chain, the use for laboratory experimentation does not entail ethical problems, as with other animal models.

**Figure 1:**
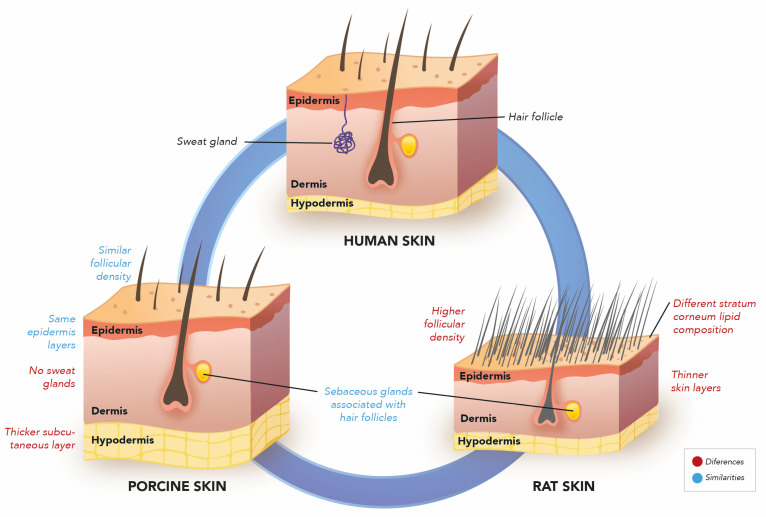
Representation of the main histological differences and similarities between porcine, rat, and human skin. Own authorship. Created with Adobe Illustrator^®^, version 27.7.

The ideal age for using porcine skin in topical penetration experiments is approximately six months to obtain a better similarity with human skin structures (Figure 1) [65]. Still, most studies in the literature do not specify this data [66,67]. The resemblance to functional human skin includes the thickness of the epidermis and dermis, the follicular structure, the density of blood vessels, as well as the cellular components [68,69], such as the presence of structures such as Langerhans cells and rete ridges [70]. The epidermis is avascular for both skins. Pig skin has an epidermis like human skin, with a comparable thickness (for pigskin, 30–140 mm and human skin, 50–120 mm). The epidermis of human and porcine skins consists of four layers: stratum basal, stratum spinosum, stratum granulosum, and stratum corneum. In both models, the epidermis and dermis are separated by a basement membrane. The rare lamina and the dense lamina of the basement membrane are clearly visible, together with the anchoring fibrils of the dermis and those of the hemidesmosomes in the plasma membrane of the cell [67,71].

The next layer, the dermis, has collagen and elastin fibers. For both species, the dermis is divided into a papillary layer and a reticular layer. Porcine dermal collagen is biochemically like human dermal collagen. The next layer, the hypodermis, is thicker in pigs than in humans. As for appendages, pig skin does not have eccrine glands, and apocrine glands are distributed over the skin’s surface. In addition, they have less vascularization compared to human skin (Figure 1). The number of hair follicles in human and porcine skin is similar. Hair follicles in humans and pigs are accompanied by sacculated sebaceous glands [71,72].

Another prominent characteristic of similarity between human and porcine skins lies in the density of hair growth (for porcine, ~20 hairs/cm^2^; for human skin, 14–32 hairs/cm^2^) [68]. This feature is crucial when follicular drug permeation is considered. Nevertheless, as mentioned before, human skin contracts after excision. Therefore, experimentally, the human skin model underestimates hair follicle accumulation, i.e., the skin contracts, and when stretched again for mounting in the diffusion cell, the multiple elastic fibers around the hair follicle remain contracted, which reduces the follicular reservoir by up to 90%. [73]. Such a problem of follicular muscle contraction is not reported for porcine skin as it is for human skin. Yet, regarding follicular contraction, the porcine ear skin model may probably be the most advantageous model, as the skin is not excised but attached to the cartilage, which inhibits such a contraction. All these characteristics have led to the wide use of the porcine skin model for in vitro studies to evaluate the follicular penetration of drugs [74].

Accordingly, the applicability of porcine skin in studies that evaluate the follicular delivery of drugs has already been extensively reported [23,26,75,76,77], with evidence of selective hair follicle targeting [78].

Figure 1 summarizes the structural and compositional differences and similarities between rat and porcine skin compared to human skin.

Finally, it should be noted that, regardless of the skin model to be used in studies involving follicular penetration of nano-entrapped drugs, depending on the disease to be managed, the physiological alteration it causes in the skin can also influence the behavior of the formulation, and the results obtained in vitro cannot reflect what would occur in an in vivo situation. This is the case of diseases that affect acne-prone skin, for example, in which the hair follicles may be clogged with excessively produced sebaceous content.

Indeed, a protocol has been recently proposed to mimic the sebaceous skin submitted to the application of some lipid nanoparticles [79]. In such a protocol, the skin was massaged with a mixture of sheep tallow and vegetable oil preceding the permeation experiments. In this adapted model, although the nanoparticles targeted the clindamycin delivery to the hair follicles in normal porcine skin compared to the free drug control when the sebaceous content obstructed the follicles, this effect was nullified.

Confirming this evidence, nanostructured lipid carriers containing the drugs used to treat hidradenitis suppurativa (clindamycin phosphate and rifampicin) were investigated using this protocol for sebaceous skin. In this case, even mimicking the sebaceous condition, the nanostructured lipid carriers accumulated in the openings of the hair follicles, not changing the amount of accumulated clindamycin compared to the regular skin model, significantly increasing rifampicin uptake in these structures by 12 times [22]. This difference between the two studies can be explained by the different composition of the lipid nanoparticles, which in the first case, had a positive superficial charge [79] and, in the second case, a negative charge [80]. Thus, not only the skin type is relevant for studying hair follicle targeting, but also some conditions of this structure relevant for the specific study design, e.g., as previously mentioned, dry or wet and with low or high sebum content.

## 3. Methods Involving the Quantification of Nano-Entrapped Drugs

The first methods presented in this section involve quantifying the nano-entrapped drug after performing the in vitro skin permeation assay. These methods do not show the location and interaction of nanoparticles after application to the skin. However, they can generate reliable data regarding the impact of nanoparticles in targeting the drugs they encapsulate. In this section, the methods of differential stripping, hair follicle occlusion, and punch technique will be presented, which are the ones that have produced the most data regarding the follicular penetration of nano-entrapped drugs.

An important experimental limitation of all these methods is the selective drug quantification following skin extraction, as the skin is a complex matrix that provides several biological interferents that can interfere with the analysis [80,81,82]. As a rule, chromatographic methods are used to fulfill the selectivity requirement. Usually, the most used types of detectors are UV or diode array [83,84,85,86,87], fluorescence [88,89], and mass spectrophotometry [90,91,92], depending on the nature of the analyzed drug.

### 3.1. Differential Stripping

Differential stripping is a derivation of the tape stripping technique, which uses a certain amount of adhesive tape that is successively applied to a region of the skin that has undergone the skin permeation process for separation of the stratum corneum and subsequent analysis of the drug accumulated in this first layer of the epidermis [59,93]. This technique (tape stripping) has been used in vivo and in vitro both in humans, mice, and porcine skin over the years [94,95,96].

**Figure 2:**
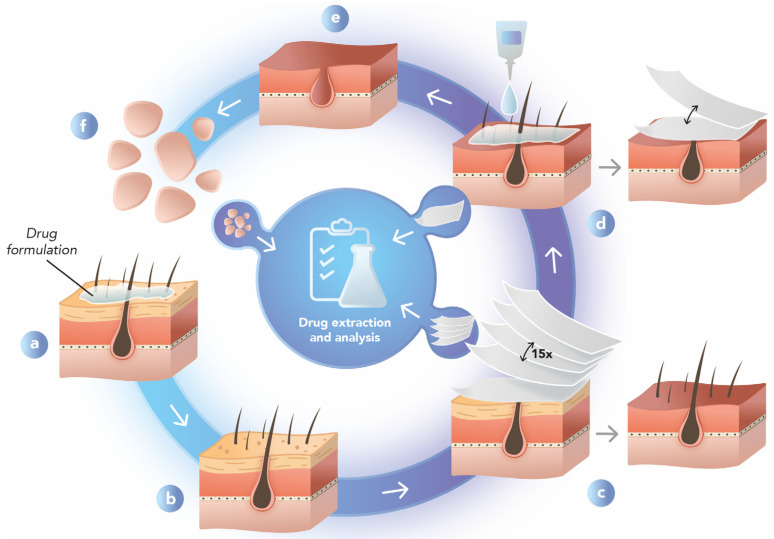
Schematic representation of the differential stripping method. (**a**) Skin is treated with a topical drug formulation; (**b**) the skin is cleaned and dried; (**c**) the stratum corneum is removed with 10–15 adhesive tapes, which are taken to drug extraction and analysis; (**d**) the follicular casts are removed with one or two drops of cyanoacrylate glue and an additional tape, which is taken to drug extraction and analysis; and (**e**) the remaining skin without the stratum corneum and hair follicles is (**f**) cut in small fragments and taken to drug analysis. Own authorship. Created with Adobe Illustrator^®^, version 27.7.

Differential stripping was initially described by Lademann et al. [97] and consists of introducing a step to remove the hair follicles after removing the stratum corneum from the skin using adhesive tapes. After applying a formulation on the skin for a predetermined experiment time, the formulation is removed and the skin is cleansed, dried, and placed on a flat surface. The stratum corneum layers are removed with about 10–15 pieces of adhesive tape in the same area with duly applied constant pressure and speed of application, named tape stripping (Figure 2). Afterward, a drop of cyanoacrylate glue is dripped by placing tape over the glue and pressing lightly for approximately 1–2 min to ensure the polymer is completely dry. The same procedure can be repeated to guarantee that the entire contents of the hair follicle are removed from the surface of the skin under study [98]. In the end, the pieces of tape containing the stratum corneum or the follicular casts are collected, and a liquid drug extraction is performed using extractor solvents. The solvent used is previously chosen through solubility studies for extracting the substance and the technique used for extraction. Then, the samples are taken for analytical analysis [15].

This methodology not only differentiates the path by which the drug reaches the deeper layers of the skin (viable epidermis and dermis)—either by permeation through the stratum corneum or penetration through the hair follicles—but also allows a comparison between formulations. As an example, such a protocol showed caprolactone nanocapsules improved latanoprost accumulation in hair follicles when topically applied to the skin and massaged, delivering 30% more drug to these skin structures than the control solution [98]. It further demonstrated that some chitosan nanoparticles doubled the minoxidil accumulation in the hair follicles in comparison with a control solution of the free drug (5.9 ± 0.6 μg/cm^2^ vs. 2.9 ± 0.8 μg/cm^2^) [76].

Furthermore, the performance of different-sized nanosystems could be compared, with lipid nanosystems of 500 nm resulting in greater penetration of entrapped propranolol (19.5 ± 0.6 μg cm^−2^) compared to some 900 nm particles (12.7 ± 0.6 μg cm^−2^). Indeed, particle size is likely to impact the interaction between the nanoparticles and the stratum corneum [99]. The differential stripping technique was also demonstrated upon topical application of some 320 nm nanoparticles containing a dye, and a depot in the hair follicles for up to 10 days was created, while the nonparticulate form could only be detected for up to 4 days [100]. This finding is specifically helpful for some diseases that affect the hair follicles and need a long-term drug delivery to be treated or even diseases that affect the deeper layers of the skin, and that a drug reservoir in the hair follicles would allow for more prolonged treatment since the drug deposited in these structures tends to have a radial permeation to other layers of the skin over time.

Another fundamental approach that this type of study can show is the concomitant delivery of two co-encapsulated drugs for treating some skin diseases. In this way, nanostructured lipid carriers containing latanoprost and minoxidil showed to preferentially deposit in hair follicles, causing a considerable increase in the penetration of the two drugs in comparison with the control (composed of the free compounds), benefiting the topical treatment of alopecia [25]. A similar finding was recently obtained by lipid nanoparticles co-entrapping the antibiotics clindamycin and rifampicin, designed for the treatment of severe infections of the hair follicles, called hidradenitis suppurativa [22].

Yet some parameters are crucial for obtaining success with the employment of this technique, e.g., the quality and quantity of cyanoacrylate glue used to remove the follicular casts must be standardized to remove all follicular contents before the drug is recovered from the remaining skin. Similarly, the previous step of tape stripping must ensure that almost 100% of the stratum corneum is separated to avoid misinterpretations.

Performing the differential stripping technique also allows the calculation of the “follicular targeting factor”. This factor determines the ratio between the amount of drug accumulated in the hair follicles by the total amount of drug that penetrated all layers of the skin (stratum corneum + hair follicles + remaining skin), as described in Equation (1) [79]:

FT = HF/TS(1)

where FT is the follicular targeting factor, HF is the amount permeated into the hair follicles, and TS is the amount of drug permeated into the total skin.

In practical terms, this factor can compare the formulation’s potential for targeting the hair follicles instead of only considering the effect of a greater or lesser drug entry. Such a factor is extremely useful in comparing different formulations, e.g., one formulation containing a penetration enhancer can provide a higher penetration into the hair follicle but concomitant to a higher penetration in all the other skin layers, while another formulation can provide lower follicle retention than the first but a much lower penetration into the other layers. In this case, FT would be higher for the latter, meaning a hair follicle targeting that could prevent adverse effects.

Such a “follicular targeting factor” is also useful in determining the influence of other parameters on the follicular accumulation process besides formulation characteristics, which has been demonstrated in vitro when the same nano lipid systems could deliver 89% clindamycin to the hair follicles but only 17% of rifampicin, showing that drug solubility characteristics may play a critical role in this targeted release effect [22]. Such a factor also facilitates data comparison. In another study, a commercial formulation of clindamycin targeted 25.6 ± 9.6% of the drug to the hair follicles, while chitosan nanoparticles increased follicular deposition to 52.9 ± 20.5%, and hyaluronic acid nanoparticles almost tripled this drug accumulation (77.0 ± 8.6%) [17].

The follicular targeting factor can also differentiate nanoparticles of different sizes. Poly-ε-caprolactone polymeric nanoparticles containing 180 nm spironolactone provided delivery of the drug to the hair follicles of about 40%, five times more than the free drug solution. Furthermore, polymeric poly-ε-caprolactone nanoparticles of 126 nm nearly doubled the follicular targeting of spironolactone compared to the control. However, the smallest nanoparticles did not differ from the control in terms of drug-targeting ability [23].

### 3.2. Hair Follicle Occlusion

The hair follicle occlusion method indirectly measures the importance of the hair follicles’ penetration pathway for drug permeation from different formulations.

**Figure 3:**
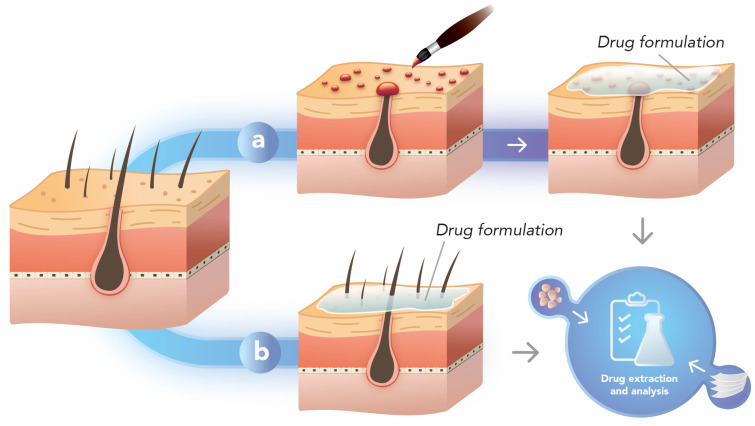
Schematic representation of the hair follicle occlusion method. (**a**) The hair follicles are occluded with enamel or resin, and the skin is then treated with a drug formulation, followed by drug extraction and quantification from the skin layers. (**b**) The results of drug retention in stratum corneum and remaining skin are compared to those from the non-pretreated skin. Own authorship. Created with Adobe Illustrator^®^, version 27.7.

The hair follicle occlusion technique consists of applying enamel to each follicular orifice to selectively block the hair follicles (Figure 3). The method requires that two cutaneous permeation experiments be implemented with the same formulation so that in comparing the results, the impact of the lack of the follicular route on the permeation of the drug from a given formulation is indirectly evaluated. In other words, it is possible to selectively analyze the penetration of substances through the skin by the intercellular and follicular pathways [101].

The type of material that blocks the follicular cavities must do so adequately. Notably, the standard enamel has shown secure follicular sealing, while solvent-free nail varnish was not able to prevent follicular penetration in an earlier conducted study [102].

The method was first applied in vivo with a caffeine formulation [101]. The formulation was applied on the thorax of the volunteers, and two experiments were carried out; the first consisted of applying the formulation to the skin with the obstructed follicles, and the second experiment was carried out one week after the first experiment with the nonobstructed hair follicles. Blocking hair follicles showed a significant difference between interfollicular and follicular penetration of topically applied caffeine. In this case, the drug was not nanostructured, but it was found that caffeine penetration via hair follicles was faster and more remarkable compared to other routes [101].

This experiment with caffeine was repeated in vitro using diffusion cells and it corroborated the previous study. Caffeine reached the receptor compartment of the diffusion cell with open hair follicles more quickly than in the case of the experiment with closed hair follicles [103]. Similarly, just five minutes after the topical application of minoxidil foam, minoxidil was detected in blood samples when follicles remained open. In contrast, with closed follicles, it took 30 min [104], demonstrating the importance of this pathway for minoxidil delivery.

This relevance of the appendageal pathway for drug penetration is evidenced by the use of polymeric nanoparticles since, for the commercial formulation of clindamycin, the closure of hair follicles did not significantly change the amount of clindamycin that reached the stratum cornea and remaining skin. For chitosan nanoparticle formulations, the closure of follicles resulted in a significant increase in penetration of the drug into the stratum corneum and a significant reduction in the drug penetration into the rest of the skin [26].

Hair follicle occlusion has proven to be the preferred way to determine the role of the follicular route on drug delivery by nanostructured lipid carriers [79]. Confirming this, nanostructured lipid carriers containing clindamycin and rifampicin demonstrated a follicular preference after comparing closed and open hair follicles versus free drugs. In other words, when the nanostructured lipid carriers were applied on the skin with the follicles blocked, the drug is prevented from interacting with the follicular region, and thus, the dynamics of penetration are modified, causing a penetration in the stratum corneum to occur [22]. Accordingly, through the follicular blockage, doxorubicin-loaded liposomes resulted in a significant reduction in the extent and intensity of fluorescence observed within the skin layers, evidencing that hair follicles were the main permeation route used by liposomes [105]. In contrast, ibuprofen nanoparticles demonstrated that the hair follicle plays less than 5% of the role in the total penetration of the nanostructured drug into the skin, as the levels of ibuprofen in the skin and the receptor compartment of the apparatus achieved were not significantly different when hair follicles were open or closed [106].

The hair follicle closure technique represents an adequate in vitro method for obtaining critical information in technological developments. However, this technique has some limitations related to the guarantee that all follicles are entirely sealed and to the interference that the enamel or varnish can pose in the quantification of the drugs.

### 3.3. Punch Test

**Figure 4:**
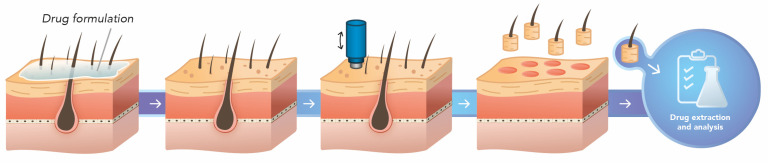
Schematic representation of the punch biopsy technique, which specifically removes each hair follicle followed by drug extraction and quantification. Own authorship. Created with Adobe Illustrator^®^, version 27.7.

The punch method has its origin and basis in a follicular unit removal technique for hair transplants. Through this technique, the entire follicular structure is removed from a location that remains viable and, thus, is reimplanted in the diseased region [107]. Thus, an optimized way to quantify drugs directly in hair follicles is through a biopsy of each hair follicle through the punch test. The study is based on applying the test formulation on the skin for a determined time. Then, each hair follicle is collected using a typically 1 mm punch; the hair shaft is cut 1 mm above the skin’s surface (Figure 4). Samples can be collected in individual Eppendorf tubes or put together in only one tube. The drug is then quantified according to assays previously validated [107].

This technique consists of using a biopsy device. However, it requires extreme attention because it is practiced manually, removing one by one the follicular units with several samples of at least 15 follicular units. The crucial points of the punch test are the difficulty in removing each hair follicle as well as the need for a more accurate and sensitive analytical method for drug quantification in small concentrations, as when each hair follicle is analyzed separately. With the biopsy, the drug is quantified in the entire follicular unit [98], making the punch method sometimes more precise than the differential stripping method.

Such a method verified the follicular penetration of polymeric micelles containing retinoic acid compared to the commercial formulation Retin-A^®^ Micro in human skin [107]. The results showed the most significant follicular deposition of the drug in the follicles was obtained with the nanoformulation (10.4 ± 3.2% vs. 0.6 ± 0.2% of the applied dose, respectively). In general, biopsy studies provide a targeted analysis of the behavior of the formulation in hair follicles [108].

## 4. Qualitative Methods

Different from quantitative methods, in which the follicular deposition of nanostructures is determined considering the quantification of the nano-entrapped drug, qualitative methods usually show a microscopic visualization of the exact nanoparticles’ location after skin application.

In general, these techniques guarantee an explanation of the penetration mechanism or even infer important data on the spatial distribution of the drug inside the different layers of the skin [109]. These studies can be carried out in parallel with the quantitative studies for a more complete picture of the mechanism of follicular-targeted drug delivery provided by nanoparticles.

These methods depend, of course, on imaging techniques like fluorescence microscopy, confocal microscopy, or tomography. These nondestructive techniques can be applied both in vitro and in vivo.

Fluorescence microscopy is based on a laser excitation source, which can be single-photon or two-photon [110]. Some biocompatible nanoparticles composed of ABA triblock copolymer PEG5K-b-oligo-(desaminotyrosyl-tyrosine octyl ester suberate)-b-PEG5K were used for the preparation of TyroSpheres for follicular administration of adapalene and were displayed in vivo within the hair follicle after being labeled with a fluorescent dye (Nile red) [111].

**Figure 5:**
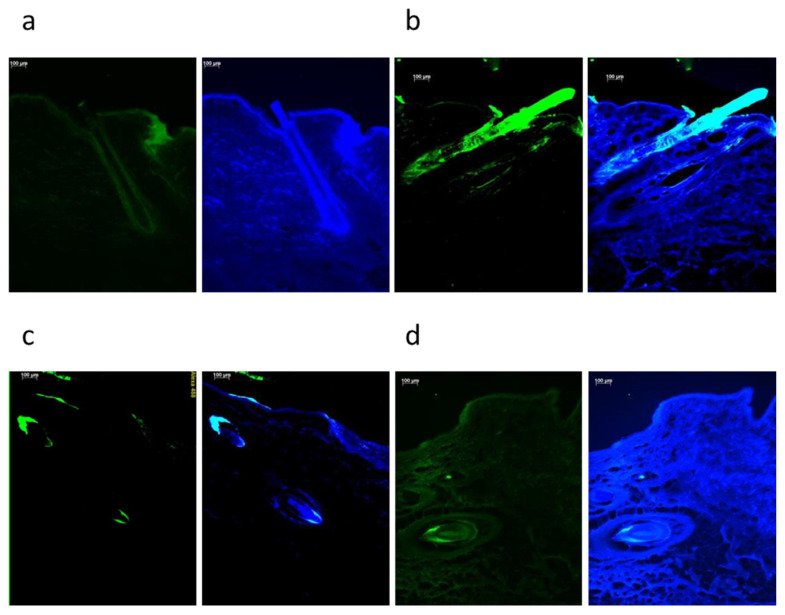
Representation of qualitative methods. Fluorescence microscope images of porcine skin sections after application of thiolated nanoparticles (**a**), sodium fluorescein (**b**), PEGylated 750 Da nanoparticles (**c**), and PEGylated 5000 Da nanoparticles (**d**). Scale bar 100 μm. The images are reproduced from [35] with permission provided by Copyright Clearance Center’s RightsLink^®^ (order number: 5590291425611).

This technique also helped our understanding of the mechanisms of follicular penetration of nanoparticles functionalized with methoxy polyethylene glycol maleimide (PEG). PEG 5000 Da functionalized nanoparticles penetrated deeper into hair follicles than PEG 750 Da functionalized ones. In this study, the fluorescence analysis was crucial to concluding that PEGylation can increase nanoparticle-targeted delivery into hair follicles [35]. The images obtained in such studies are reproduced in Figure 5 below.

Confocal laser scanning microscopy is recognized for being noninvasive, i.e., the technique enables optical cuts of the analyzed tissues, allowing the nonprocessing of samples with vitro assays. Furthermore, the technique can be adapted for in vivo analysis. In addition, higher-resolution images are obtained compared to fluorescence microscopy [112]. For this reason, even though the equipment is more expensive than fluorescence microscopes, its use is more common for local analysis of labeled nanoparticles [113]. This technique emits a laser source in a monochromatic single beam, exciting the fluorescent markers. The images produced make it possible to characterize the skin in depth in various focal planes [15,114]. The principle of the technique causes differentiation of the light coming from different planes of the specimen to occur, thus being able to capture images of samples in complex biological tissues, such as skin, with high resolution. Thus, this technique has been employed in different studies and has helped to understand the behavior of different nanosystems [15,115].

Fluorescence and confocal laser scanning microscopy are performed after labeling the nanostructure with a fluorescence substance before application on the skin [116]. The techniques’ most-used fluorescent markers are fluorescein, Nile red, and 5-bromodeoxyuridine. These components are generally placed in nanostructured systems during their preparation to characterize the permeation profiles of these fluorescent markers through the skin appendages [109]. The red fluorescence labeling of three polystyrene nanoparticles of different sizes allowed us to see the location of the nanoparticles in the stratum corneum and hair follicles without penetrating the epidermis/dermis. In addition, using variables, we observed that changing the barrier with strip removal and changing the incubation temperature did not induce deeper penetration [117].

The Nile red marker is used to locate and quantify lipids, particularly neutral lipid droplets within cells. Nile red undergoes increased fluorescence and significant absorption and emission changes to blue in nonpolar environments but is almost nonfluorescent in water and other polar solvents. Images obtained by confocal laser-scanning microscopy also determined that polymeric micelles loaded with cyclosporin A labeled with Nile red were preferentially deposited between corneocytes and in the intercluster regions (i.e., between clusters of corneocytes) with more profound skin penetration in these structures [118]. Similarly, the micelle formulation with benzoyl peroxide also demonstrated approximately threefold higher drug deposition (3.63 ± 1.23 µg·cm^−2^) in porcine skin than a commercial gel preparation (1.36 ± 0.77 µg·cm^−2^), proving to be more effective than the conventional commercial gel preparation to deliver the drug to the skin. Here, the confocal microscopy images confirmed the penetration of Nile red-labeled nanoparticles into the hair follicles [119].

The rhodamine 6G marker is commonly used as an in-water marker to determine the rate and direction of flow and transport. Rhodamine dyes fluoresce and can therefore be detected easily and inexpensively with fluorometers. The marking of the nanosystem and the technique of confocal laser-scanning microscopy provides an analysis of the location of the nanosystem in each layer of the skin under study. Dermal penetration nanostructured lipid carriers containing rhodamine-labeled 17-α-estradiol showed that in cross-sections, the fluorescence indicates that the nanostructured lipid carriers’ formulation accumulates less on top of the skin and more in the hair follicles [120]. The aqueous solution of rhodamine 6G was distributed in the stratum corneum and the shallow part of the hair follicles. In contrast, the suspension of PLLGA nanoparticles encapsulated in rhodamine 6G was distributed in the stratum corneum and the deep part of the hair follicles [121].

Fluorescein markers are fluorescent and mostly used in research with biological samples due to their high absorptivity, excellent fluorescence quantum yield, and good solubility in water. Fluorescein isothiocyanate-labeled bovine serum albumin hydrogel nanocarriers loaded with the model drug and fluorescent dye tetramethylrhodamine-dextran were applied topically to porcine ear skin. Confocal laser-scanning microscopy shows a slightly but statistically significant deeper follicular penetration of fluorescent signals corresponding to fluorescent dye tetramethylrhodamine-dextran instead of fluorescence corresponding to fluorescein isothiocyanate-labeled particles [122].

Confocal microscopy also enables the analysis of hair follicles’ role as reservoirs for dermal drug delivery, as in the case of polystyrene and poly-(methyl methacrylate) nanoparticles that were in the skin “grooves” and around hair follicles [123].

Also, confocal microscopy can observe the depth with which the nanoparticles can reach the follicular casts upon topical application. A thermoresponsive nano gel labeled with indodicarbocyanine (189 nm) showed a significant increase in mean follicular penetration of the carrier to a depth of 298.8 ± 85.8 μm after incubation at 37 °C compared to samples incubated at 21 °C and 32 °C with mean follicular penetration depths of 202.7 ± 81.7 μm and 219.4 ± 52.9 μm, respectively [78]. Similarly, curcumin-loaded lipid nanoparticles demonstrated that these nanosystems show penetration reaching 235 μm ± 48 μm in hair follicles [124].

Employing the laser scanning confocal microscopy technique, an increase in the amount of permeation of the hair follicles is observed through the evaluation with lipophilic dye and with the use of the vehicle (surfactants-propylene glycol) in the application of lipophilic dyes in fresh human scalp skin [125].

In each phase of development, the ability to predict the proper condition of their use ensures the credibility of new nanoformulations. To demonstrate this, polymeric nanoparticles of poly(q-caprolactone)-block-poly-(ethylene glycol) containing minoxidil were applied to the skin of a guinea pig. The confocal microscopy technique demonstrated that the nanoparticles containing solutes penetrated mainly via bypass pathways, such as hair follicles, resulting in the absorption of solutes through the skin [126].

In some cases, the technique made it possible to determine the permeation of nanoparticles in the skin over time. As demonstrated, at 4 h they were more concentrated on the skin’s surface as fluorescence was more significant in the stratum corneum. Then, after 6 h, total fluorescence decreased in the stratum corneum and increased in the follicular cells, indicating the movement of the nanoparticles [127].

In the construction and development of new nanosystems, preferentially for studies of the follicular structures, an evaluation of the new nanosystem versus a formulation already used in the market shows that this new technology guarantees its therapeutic potential. Skin deposition of tacrolimus using the optimized 0.1% micelle formulation after application for 4, 8, 12, and 24 h was significantly greater than that from Protopic^®^ (0.1% *w*/*w*; tacrolimus ointment) at each time point. The maximal tacrolimus deposition was achieved after 24 h (11.51 ± 3.05 μg/cm^2^ and 0.75 ± 0.23 μg/cm^2^ for micelles and Protopic^®^ 0.1% *w*/*w*, respectively). The preferential deposition of micelles into the hair follicle was also confirmed by confocal laser-scanning microscopy [128]. Similarly, the micelle formulation with benzoyl peroxide demonstrated approximately threefold higher drug deposition (3.63 ± 1.23 µg·cm^−2^) in porcine skin than a commercial gel preparation (1.36 ± 0.77 µg·cm^−2^), proving it to be more effective than the conventional commercial gel preparation to deliver the drug to the skin. The confocal microscopy images confirmed the penetration of Nile red into the hair follicles [119].

Raman spectroscopy is another technique that is being used to evaluate permeation. The technique governs the principle of inelasticity of the light scattering with monochromatic characteristics in a single laser beam. Changes in the wavelength of the photons can identify the samples. An excitation or deactivation of molecular vibrations is related to the energy variations of the photons, and it provides information about the molecular structure of tissue components, with the advantage of not having to use fluorescent markers or chemical dyes. Recently, the technique of confocal Raman microscopy combines the spectral information from Raman spectroscopy with the spatial filtering of a confocal optical microscope for high-resolution chemical imaging of samples [129,130].

This method may reveal changes in the components of the skin structure while investigating drug or nanosystem permeation [131]. As highlighted, this methodology describes the skin in depth in a noninvasive way [131]. Raman spectra have been used to compare human and porcine hair follicles [132]. By applying the Raman technique, it was possible to confirm that after 30 min of permeation, retinol acetate was found at a depth of 20 μm in the stratum corneum, demonstrating that this technique can determine the location of the product in the skin in the study [133]. In this case, the drug had sufficient fluorescence to be located by the technique. In addition, imiquimod-loaded chitosan nanocapsules showed dynamic transdermal penetration and took about 50 min to penetrate the stratum corneum, and 24 h after transdermal administration, the drug was in the inner layers of the skin [134].

The combination of techniques also favors a better development of nanoformulations. Detailed information on molecular composition can be obtained for well-defined regions by combining confocal and Raman microscopy. This promotes a detailed study of specific skin structures (sweat canal, sebaceous gland, dermal capillary), which supports the development of nanoformulations for follicular treatment [135].

Qualitative methods are more advantageous than other techniques because they visualize permeative processes. Fluorescence methods face a slight difference in resolution compared to other methods and the need for an extensive cooling system and obtaining lasers. The confocal technique has overcome these resolution issues due to its high complexity in marking samples and specific detection of nanosystems in follicular structures. However, regarding permeation studies, it is observed that this technique is not dynamic and reports only a fraction of the permeation process in question in addition to being semi-quantitative determinations. The Raman methodology has been widely used for promoting a specific situational state of the nanoparticles in the matter of depth in the skin. Nevertheless, during processing, there is an increase in sample temperature, which can destroy them. Furthermore, it has a substantial analysis limitation due to interference in the deep layers of the skin.

Table 1 presents some examples of studies evaluating the follicular penetration of nanostructured drugs using either quantitative or qualitative methods.

## 5. Conclusions

In conclusion, for in vitro studies, human skin can possibly be replaced by other types of skin, mainly porcine skin, due to its availability, avoidance of ethical concerns, and mainly because the hair follicles resemble the human anatomy conditions after extraction more than the human in vitro model itself. Thus, porcine ear skin has been widely used as an alternative in studies of follicular penetration. In general, for assessing follicular penetration, the choice of test methodological conditions must be consistent with the pathophysiology of the skin to be treated to obtain consistent results. Several tools have been developed in this regard, such as differential tape stripping, the follicular-blocking method, and the punch biopsy technique, in addition to the microscopic techniques of fluorescence, confocal, and tomography, which, if properly used, can finally make it possible to take advantage of the potential of nanotechnology for hair follicle-targeted drug delivery.

## Figures and Tables

**Table 1:** Examples of studies evaluating the follicular penetration of drugs using different methodologies.

Type	Technique	Skin Model	Findings	References
Quantitative methods	Differential stripping	Porcine	30% more drug in hair follicles from nanoparticles compared to control solution.	[98]
		Porcine	Doubled the minoxidil accumulation in the hair follicles. Drug are deposited in the hair follicles for up to 10 days.	[76,100]
	Occlusion of the hair follicle	Porcine	Blocking hair follicles showed a significant difference between interfollicular and follicular penetration of topically applied caffeine.	[101]
		Human	Caffeine reached the receptor compartment of the diffusion cell with open hair follicles more quickly compared to closed hair follicles.	[103]
		Human	In just 5 min, minoxidil was detected in blood samples when follicles remained open compared to 30 min with closed follicles.	[104]
	Punch test	Human	The most significant follicular deposition of the drug in the follicles was obtained with the nanoformulation.	[108]
Qualitative methods	Fluorescence microscopy	Porcine	PEG 5000 Da functionalized nanoparticles penetrated deeper into hair follicles compared to PEG 750 Da functionalized ones.	[35]
	Confocal laser scanning microscopy	Human	The technique allowed seeing nanoparticles in the stratum corneum and hair follicles without penetrating the epidermis/dermis.	[117]
		Porcine	Micelle promotes threefold higher drug deposition than a commercial gel preparation.	[119]
		Porcine	Nanostructured lipid carriers’ formulation accumulates less on top of the skin and more in the hair follicles.	[120]
	Raman spectroscopy	Porcine	The drug was in the inner layers of the skin.	[135]

## Data Availability

No new data were created.
